# ARTIFICIAL INTELLIGENCE IN COLONOSCOPY: EVALUATION OF ADENOMA DETECTION RATE AND PERFORMANCE CHARACTERIZATION

**DOI:** 10.1590/S0004-2803.24612024-121

**Published:** 2025-12-01

**Authors:** Carolina Roos MARIANO DA ROCHA, Sophia Andreola BORBA, Thales Tomaz RICHINHO, Leonardo Wagner GRILLO, Fernando Comunello SCHACHER, Rafael Castilho PINTO, Fernando Herz WOLFF, Fabio SEGAL

**Affiliations:** 1 Hospital Moinhos de Vento, Unidade de Endoscopia, Porto Alegre, RS, Brasil.; 2 Hospital Moinhos de Vento, Serviço de Gastroenterologia, Porto Alegre, RS, Brasil.; 3 Hospital Moinhos de Vento, Serviço de Coloproctologia, Porto Alegre, RS, Brasil.

**Keywords:** Colonoscopy, artificial intelligence, colorectal cancer, cancer screening, Colonoscopia, inteligência artificial, câncer colorretal, rastreamento

## Abstract

**BACKGROUND/OBJECTIVE:**

Colonoscopy is a known tool for diagnosing precursor lesions of colorectal cancer. The use of AI has the potential to enhance the detection of these lesions. The aim of this study is to compare the adenoma detection rate (ADR) in colonoscopies performed with and without AI software.

**METHODS:**

An observational study was conducted in the endoscopy department with the contribution of the gastroenterology, coloproctology and pathology services, all from a tertiary hospital in the southern region of Brazil**.** A total of 305 patients undergoing screening colonoscopy were evaluated. Patients were scheduled for colonoscopy through random assignment to procedure rooms with high definition conventional colonoscopy (CC) or with “Cadeye” (CADe) system. The metrics associated with patients, the procedure and polyps’ features were recorded.

**RESULTS:**

Of 305 colonoscopies, 112 were in the CADe system and 193 in the CC. 470 polyps were detected. The overall ADR was 53.8% and the overall polyp detection was 74.8%. There was no difference in the ADR between CC and CADe (57% vs 48.2%, respectively. *P*=0.138).

**CONCLUSIONS:**

Although data argue for an increase in ADR with AI systems, our study did not show difference between conventional colonoscopy or AI. These findings suggest that, in settings with high ADR, the added benefit of AI may be limited.

## HIGHLIGHTS

• The incidence of colorectal cancer has been increasing in the past decades, leading to the necessity of new approaches to improve adenoma detection rate in order to increase diagnoses. The introduction of artificial intelligence (AI) softwares during colonoscopies might improve detection of early lesions according to previous data. The present study evaluated the adenoma detection rates in patients who underwent screening colonoscopies, divided into two subgroups: one with the conventional technique, and the other using a specific AI system. However, no significant difference was found between these two modalities in the adenoma detection rate.

## RESUMO

**Contexto:**

Colonoscopia consiste em uma ferramenta bem estabelecida para diagnóstico de lesões precursoras de câncer colorretal. O uso de inteligência artificial (IA) possui o potencial de melhorar a detecção dessas lesões. O objetivo deste estudo é comparar a taxa de detecção de adenomas em colonsocopias realizadas com e sem softwares de IA.

**Métodos:**

Um estudo observacional foi conduzido no departamento de Endoscopia, com a contribuição dos serviços de Gastroenterologia, Coloproctologia e Patologia, todos de um hospital terciário na região Sul do Brasil. 305 pacientes submetidos a colonoscopias de rastreamento foram avaliados. Os pacientes eram agendados randomicamente para a realização de colonoscopia Os pacientes foram agendados para realização de colonoscopia de forma aleatória para salas de procedimento com colonoscopia convencional (CC) de alta definição ou com sistema “Cadeye” (CADe). Os parâmetros relacionados aos pacientes, ao procedimento e às características dos pólipos foram registrados.

**Resultados:**

Das 305 colonoscopias, 112 delas foram realizadas com o sistrema CADe e 193 com o sistema CC. 470 pólipos foram detectados. A taxa de detecção de adenomas global foi 53.8% e a taxa de detecção de pólipos geral foi 74.8%. Não houve diferença na taxa de detecção de adenoma entre os sistemas CC e CADe (57% vs 48.2%, respectivamente. *P*=0,138).

**Conclusão:**

Embora os dados apontem um aumento da taxa de detecção de adenomas com o uso de sistemas de IA, nosso estudo não demonstrou diferenças entre a colonoscopia convencional ou com uso de IA. Esses achados sugerem que, no cenário de altas taxas de detecção de adenoma, o uso de IA pode não ser tão relevante.

## INTRODUCTION

Colorectal cancer (CRC) is the second leading cause of cancer-related mortality worldwide, reaching almost one million deaths in 2020, with a predicted increase to 1.6 million deaths over the next 20 years in developed countries^1^. The methods to prevent CRC cases include not only addressing modifiable risk factors but also conducting population-wide screening colonoscopies based on age, previous family history, and other specific characteristics^2^ ^,^ ^3^.

The standard parameter used to assess the quality of a colonoscopy is the adenoma detection rate (ADR), which measures the proportion of patients who underwent screening colonoscopy in whom had at least one adenoma detected^4^. Guidelines recommend a general ADR target of 25%, comprising 20% for women and 30% for men^5^. ADR can vary depending on factors related to the endoscopist, the patient, and the equipment used^4^ ^,^ ^6^, resulting in a proportion of polyps that go undetected during the examination^7^. Given that mortality has not changed significantly in recent decades, coupled with the increasing incidence of CRC in younger patients^8^, the optimal detection of pre-malignant colorectal lesions remains a highly sought-after objective among colonoscopists. In this context, artificial intelligence (AI) systems have emerged as a potential tool to overcome human limitations, reduce inter-operator variability, enhance diagnostic accuracy, and assist in rapid and precise therapeutic decisions^7^.

AI is a complex concept that involves the ability of computer systems to self-learn and solve problems autonomously. It enables machines to better recognize human shortcomings and address these weaknesses, thereby improving productivity and effectiveness^9^. First descriptions of AI as an additional medical tool emerged in the 1960’s^10^ and it has been increasingly applied in the endoscopy field. However, the association of AI with screening colonoscopies remains a contentious issue, with conflicting studies and a lack of robust evidence demonstrating an increase in the ADR^11^. While physicians, in general, believe that AI improves the quality of colonoscopies in some way, some researchers emphasize that AI is still in its early stages in the medical field, with insufficient data to determine definitive changes in our clinical management up to the present moment^12^.

High-tech devices are undoubtedly here to stay, but endoscopists must decide how to judiciously use them. Therefore, the aim of the present study is to compare the ADR in screening colonoscopies performed with or without an AI system to better understand its application and to evaluate the performance of the AI system in predicting polyp histology.

## METHODS

An observational, cross-sectional study was conducted in the Endoscopy department of a private tertiary hospital in the southern region of Brazil. The study included patients of both genders, aged 18 or older, who underwent colonoscopy for CRC screening between April 1, 2023, and August 1, 2023, and who agreed to participate by providing informed consent. Patients with poor bowel preparation (Boston Bowel Preparation Scale [BBPS] <6), incomplete exams (failure to reach the cecum), a diagnosis of inflammatory bowel disease, a previous diagnosis of CRC, prior colectomy, familial adenomatous polyposis syndromes, and contraindications to polypectomy were excluded. The study received approval from the institution’s ethics committee.

All exams were conducted at a single Endoscopy center. The study was an open-label, non-blinded cohort, and groups were randomly organized based on routine service exams. Simple randomization was adopted, with random assignment of patients and colonoscopists to available rooms - one of them equipped with AI technology. Five colonoscopists with at least five years of experience participated in the study, occasionally accompanied by a Gastroenterology or Endoscopy resident. All participating physicians performed exams both with and without AI assistance. All exams were conducted under anesthesia supervision.

**FIGURE 1:**
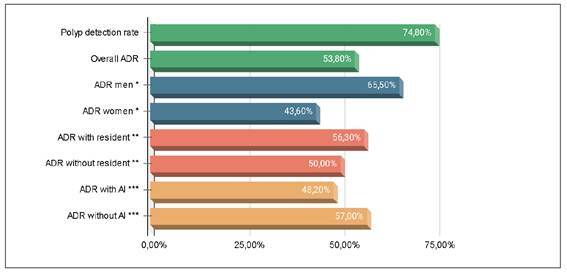
Results of the primary and secondary outcomes.

**FIGURE 2:**
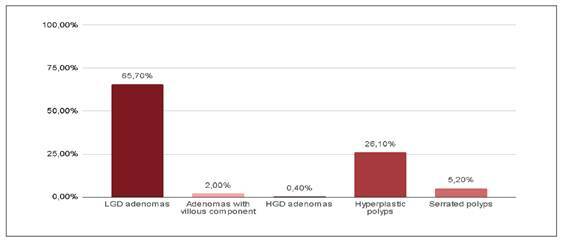
Characteristics of resected polyps. There was no statistical difference in the detection of each polyp type between conventional and artificial intelligence colonoscopies.

The exams utilized the same brand and high definition colonoscope (EC 720R, Fujifilm, Tokyo). In the AI group, the “CadEye” (CADe) system was used, which operates with a type of AI called deep learning, analyzing real-time endoscopic images during colonoscopy without requiring additional techniques such as magnification or image capture. When a suspicious polyp is detected, a detection box indicates the area where the polyp was detected, accompanied by an audible signal (Figure 1). Additionally, this system includes Computer-Aided Diagnostic (CADx) technology, providing predictive histological evaluation, suggesting the histology of polyps as neoplastic or hyperplastic (Figure 2).

ADR: adenoma detection rate. AI: artificial intelligence. *P<0.05. **P=0.281. ***P=0.138.

LGD: low grade dysplasia. HGD: high grade dysplasia.

The study compared the ADR, defined as the proportion of patients undergoing screening colonoscopy with at least one detected adenoma, between conventional colonoscopies and colonoscopies with AI. General patient and exam characteristics were evaluated in both groups: gender, age, bowel preparation quality through the Boston Bowel Preparation Scale (BBPS), withdrawal time, and the presence of a resident in the room. Polyp characteristics, including location, size, Paris classification, and histological diagnosis, were also compared between the conventional colonoscopy group and AI group.

Polyps were assessed, when possible, using chromoendoscopy (conventional or digital), described by the endoscopist based on location, size, Paris classification, Japan NBI Expert Team (JNET) vascular pattern, and Kudo pit pattern. In exams with AI, polyps were classified as hyperplastic or neoplastic based on the AI system’s impression. Polyps were excised as per the colonoscopist’s recommendation, using the most appropriate technique for each situation, and sent separately for histopathological examination. Histopathological results were later verified. Data were stored in a database created exclusively for this study.

In the AI group, the agreement between the endoscopist’s histological suggestion during the exam, the AI system’s suggestion, and the histopathological examination (considered the gold standard) was evaluated. Performance measures (sensitivity, specificity, positive predictive value, negative predictive value, and accuracy) of polyp assessment by the colonoscopist and the AI system relative to histopathological diagnosis were also conducted.

Sample size was defined to encounter a difference of 20% in adenoma detection using conventional colonoscopy and AI-based colonoscopy (20% vs 40%). Considering a power of 80% and α of 0.05, a total of 82 patients per group were necessary for the analyses. Continuous variables were described using mean and standard deviation or median and interquartile range, depending on data normality verified by the Kolmogorov-Smirnov test. Normally distributed variables were evaluated using Student’s t-test, and non-normally distributed variables were assessed using the Mann-Whitney U test. Categorical variables were described using frequency and percentage and analyzed using the chi-square test with Yates’ correction. The Kappa coefficient was used to assess the agreement among colonoscopist, AI system, and histology evaluations. *P*-values less than 0.05 were considered statistically significant.

## RESULTS

A total of 324 patients met the inclusion criteria, with 305 eligible for the study. Nineteen patients were excluded: fourteen patients were excluded due to poor bowel preparation; one due to incomplete examination; two with a previous diagnosis of inflammatory bowel disease; one with prior colectomy; and one using anticoagulation, contraindicating procedures such as polypectomy.

The mean age of patients was 57.9 years (±12.5). Of the patients, 163 (53.4%) were female. The vast majority (88.2%) had excellent bowel preparation, classified as BBPS 9. The overall median withdrawal time was 12.5 minutes (interquartile range [IQR] 10-17).

Five colonoscopists participated in the study, conducting exams with the presence of a Gastroenterology or Endoscopy resident in 183 of 305 exams (60%). A total of 112 exams were performed with AI-equipped devices, and 193 with conventional high-resolution white-light colonoscopy. There were no statistically significant differences in age, bowel preparation quality, or presence of a resident in the room between the conventional colonoscopy group and AI group (Table 1).

**TABLE 1:** Baseline characteristics of patients and colonoscopy examinations performed with and without artificial intelligence.

	With AI (n=112)	Without AI (n=193)	
Age*	58.27 (+12.12)	57.68 (+12.79)	*P*=0.902
Female gender**	47/112 (42%)	116/193 (60.1%)	***P*=0.020**
Bowel Preparation (BBPS=9)**	96/112 (85.7%)	173/193 (89.6%)	*P*=0.306
Presence of resident in the exam room **	75/112 (67%)	108/193 (56%)	*P*=0.059
Colonoscope withdrawal time (in minutes)***	11,5 (IQR 8-15.8)	13,5 (IQR 10.5-20)	***P*<0.001**

AI: artificial intelligence. *Mean+Standard Deviation (Student’s t-test). **Frequency and % (chi-square test). ***Median and Interquartile Range (Mann-Whitney test).

In total, 470 polyps were identified among the 305 patients included in the study. The overall ADR was 53.8%, with rates of 43.6% in women and 65.5% in men (*P*<0.005). There was no statistically significant difference in ADR between the groups, with ADRs of 48.2% in the AI group and 57% in the conventional colonoscopy group (*P*=0.138). No statistically significant difference in ADR was observed among the participating colonoscopists. ADR was slightly higher when a resident was present in the room (56.3%) compared to when no resident was present (50%), but this difference was not statistically significant (*P*=0.281). The polyp detection rate was 74.8%, with no significant difference between the groups.

Sessile polyps (Paris classification 0-Is) accounted for 66.7% of all polyps. Histologically, 65.7% were low-grade dysplasia adenomas, 26.1% hyperplastic, 5.2% serrated, 2% adenomas with villous components, 0.4% adenomas with high-grade dysplasia, and 0.7% of resected polyps showed evidence of neoplasia. Concerning the resection technique, 57.5% were removed via cold snare polypectomy, 32.3% with biopsy forceps, 1.7% via hot snare polypectomy, and 8.5% through mucosectomy. More than half (56.5%) of identified polyps were located in the right colon. When comparing polyp characteristics between exams with and without AI, there were no statistically significant differences in polyp size, Paris classification, frequency of hyperplastic polyps, location, or histological diagnosis.

For polyps’ histological assessment, colonoscopists described the vascular pattern using the JNET classification and the crypt opening pattern using the Kudo classification, whenever possible. The AI system provided real-time predictions, classifying polyps as hyperplastic or neoplastic based on endoscopic image characteristics. Lastly, the the final histological diagnosis was given by a pathologist, considered the gold standard. was determined by the pathologist. Concordance between the colonoscopist’s impression, the AI system’s prediction, and the histopathological diagnosis was assessed using the Kappa coefficient.

The colonoscopists’ diagnostic impression agreed with the histopathological diagnosis in 80.6% of cases, with a Kappa coefficient of 0.447, indicating moderate agreement. The AI system’s impression agreed with the histopathological diagnosis in 73.7% of cases, with a Kappa coefficient of 0.447. Performance metrics for both the colonoscopists and the AI system relative to the histopathological diagnosis are presented in Table 2. Among the polyps where there was disagreement between the AI system and the histopathological diagnosis, 24 were histologically adenomas but were classified as hyperplastic by the AI, and 7 were classified as neoplastic by the AI but were histologically hyperplastic.

**TABLE 2:** Performance measures of the artificial intelligence system and the endoscopist’s assessment relative to histopathological diagnosis.

	Artificial Intelligence	Endoscopist
Sensitivity	70.5%	85.7%
Specificity	81.6%	66%
Positive Predictive Value	90.5%	87.8%
Negative Predictive Value	52.5%	61.8%
Accuracy	73.6%	80.6%

## DISCUSSION

In the present study, we analyzed the performance of AI in the detection of adenomas in a single-center, real-life scenario involving five attending endoscopists. The findings were negative regarding a higher adenoma detection rate (either non-advanced or advanced adenomas) or sessile serrated lesions (SSL) with AI-assisted colonoscopy, similar to studies conducted in real-world settings.

Although randomized clinical trials and meta-analyses^13^ ^-^ ^15^ have demonstrated benefits with AI-assisted colonoscopy regarding higher ADR^16^ ^-^ ^21^, lower adenoma missing rates (AMR), or SSL miss rate^22^, three aspects should be emphasized: first, almost all clinical trials showed a higher ADR in the AI group. However, they had a higher withdrawal time in the AI arm - a well-known cornerstone for improving the ADR^23^ - compared to the conventional colonoscopy group^16^ ^-^ ^21^ ^,^ ^24^. The importance of withdrawal time is evident even in AI studies, as the clinical trials with higher ADR had a higher withdrawal time^22^ ^,^ ^25^. Second, the average polyp detection rate (PDR) detected in some of the previous studies was relatively low compared to ours. The high PDR was probably possible because we had a higher mean withdrawal time, used a high-definition scope, and had a mean great bowel preparation, suggesting that the use of AI may not be necessary in improving quality metrics when the parameters stated in guidelines have been followed accordingly. Third, the technologies used are from different enterprises with different machine learning tools, an aspect that may not yield reproducible results in different scenarios. Another issue that must be addressed is the fact that we had a high percentage of optimal bowel preparation in both groups, favoring the identification of polyps^26^.

Another issue evaluated in the present study is the agreement among the endoscopic assessment through the pit pattern, possible histology findings (neoplastic versus hyperplastic) given by the AI, and their correlation with anatomopathological analysis (previously defined as gold standard). The kappa coefficient was classified as moderate, indicating a poor correlation regarding the analysis in AI when compared to histological evaluation. An important aspect to highlight is that we established that the first diagnosis performed by the AI would be used as its final answer. This is important because the pattern given by the software often changes if the scope was still for a few seconds in front of the lesion.

Also, despite showing high sensitivity and positive predictive value, the AI system exhibited a low negative predictive value. Of the polyps identified where the AI did not agree with the histopathological diagnosis, 24 were tubular adenomas with low-grade dysplasia that were identified as hyperplastic by the AI system, and 7 were hyperplastic but were identified as neoplastic by the AI system. Whether the AI system made more errors in relation to initially considered hyperplastic polyps that were histopathologically adenomas is an important consideration. In addition to the need for standardization among endoscopists during polyp assessment, such as lesion cleansing and observation time, it is also necessary for AI software to be constantly updated to overcome these discrepancies.

The present study has some limitations. For a better comprehension of AI role in polyps detection and characterization, the present study was not blinded. However, lack of blindness leaves research vulnerable to investigators’ subjectivity, increasing the chances of a biased analysis. In our study, as an attempt to reduce biases, all patients were treated according to standard guidelines using objective parameters for polyps treatment (for instance, size and morphologic characteristics). Additionally, randomization also helps to reduce bias, minimizing differences between each group. Cross-sectional design also precludes stratification of higher-risk groups of having neoplastic polyps, such as obese individuals, those with a first-degree history of CRC, or smokers. 

Studies involving residents also present biases. First, residents are in different learning curves, so that their polyp detection differs substantially among them - some can miss numerous polyps, while others can misinterpret polyps or even mucosa irregularities as adenomas. For this reason, at least one more experienced doctor is always supervising residents’ exams. Nevertheless, the presence of two or more doctors can increase adenoma detection^27^, leading to an overestimation of adenoma detection that actually do not correspond to a real-life scenario. On the other hand, residents may characterize some polyps as adenomas that experts would not classify this way, increasing the chance of false positives and therefore biasing analyses.

Potential biases involving AI should also be taken into consideration, such as performance gaps. The majority of AI algorithms have been created based on populations that differ substantially from Brazilians, such as Asian individuals, who present important differences when compared to a Western population. This could lead to a misreading of the polyps’ nature and consequently to wrong assignments, distorting final statistics. Additionally, many AI algorithms were built with an important lack of data regarding specific subgroups. Black patients can be incorrectly assigned as “low risk” based exclusively on their polyps’ appearance, bringing a significant number of false negatives that influence in the final analyses.

In conclusion, we have shown the feasibility of achieving similar metrics in colonoscopy, whether using the AI software or not. The available evidence suggests that AI is a valuable tool for improving polyp detection and should not be discouraged. However, its added value may be limited, and further research is needed to fully comprehend how AI can best contribute in this context.

## Data Availability

The research data are presented within the article itself (available in the Results section, Tables 1 and 2 and Figure 1 and 2).
